# Fibroblast Growth Factor-2 and the Invasive Potential in Urothelial Malignancies of the Bladder

**DOI:** 10.7759/cureus.34147

**Published:** 2023-01-24

**Authors:** Hristo Popov, George S Stoyanov, Peter Ghenev

**Affiliations:** 1 General and Clinical Pathology, Forensic Medicine and Deontology, Medical University of Varna, Varna, BGR; 2 General and Clinical Pathology, St. Marina University Hospital, Varna, BGR

**Keywords:** tumor invasion, tumor-extracellular matrix interactions, fibroblast growth factor 2, urinary bladder malignancies, urothelial carcinoma

## Abstract

Introduction

Urothelial carcinomas represent a distinct group of malignancies with a high recurrence potential. Multiple studies have established a set of interactions between the tumor cells of urothelial neoplasms and the extracellular matrix regarding invasion and tumor progression. In the present study, we evaluated the expression of fibroblast growth factor-2 (FGF2) in early-stage urothelial carcinomas of the urinary bladder (pTa and pT1) regarding the invasive potential of these tumors.

Materials and methods

A retrospective non-clinical approach was utilized for the study. Tumor tissue sections used for the initial diagnosis were stained by immunohistochemical means with an anti-FGF2 antibody and the expression within the extracellular matrix was evaluated using a histo-score (h-score). Statistical parameters regarding tumor invasion, FGF2 expression pattern and levels, patient demographic characteristics, and disease recurrence were analyzed for significance.

Results

A total of 163 cases were analyzed, with an h-score of 110 determined as the optimal cut-off value for invasive potential regarding FGF2 expression, with a sensitivity of 75.4% and a specificity of 78.9%. No statistical correlation was established between the demographic profile of the patients and the occurrence of disease recurrence.

Conclusion

Based on our results, the study of tumor-extracellular matrix interactions in regards to FGF2 expression is a promising field, at least in urothelial malignancies of the urinary bladder, in regards to tumor invasive potential, while it remains unestablished how these interactions affect metastatic potential.

## Introduction

Urothelial carcinomas are unique among malignant neoplasms due to the clinical peculiarities of their development and progression and the lack of well-established morphological predictors of invasion, recurrence, and metastasis [1]. A recently promising area of the study of malignant tumors concerning their microenvironment is the interaction between the neoplastic process and the cell located in the tumor stroma, which is rich in different types of cells that secrete different biological substances with a protumor or antitumor effect [2].

One type of tumor stroma cells are fibroblasts, specialized mesenchymal cells with a spindle-shaped nucleus. Their role is in the regulatory process of extracellular matrix synthesis, change in connective tissue, and structural maintenance. Fibroblasts of the same or different origins show considerable heterogeneity. Different protein markers such as vimentin, smooth-muscle-actin (SMA), desmin, fibroblast-activation protein, and fibroblast-specific-protein 1 are needed for their identification and utilization [3].

The expression of these markers in different combinations is observed in various pathological conditions. These characteristics are thought to reflect the adaptation and role of fibroblasts relative to their environment.

A notable subclass of fibroblasts are myofibroblasts, which behave like fibroblasts and have contractile cytoplasmic filaments akin to smooth muscle cells, which allow them to contract [4].

Like smooth muscle cells, they express αSMA, a marker for their identification. Although the exact origin of myofibroblasts is controversial, it is generally accepted that they can originate from multiple cell lineages. These include local fibroblasts, bone marrow progenitor cells, and epithelial or endothelial cells. Local fibroblasts are thought to transdifferentiate into myofibroblasts, while bone marrow progenitor cells are derived at the site prior to transdifferentiation. Relatively recent findings indicate that emerging myofibroblasts can originate from epithelial or endothelial cells through epithelial-mesenchymal transformation [5,6].

While under physiological conditions, myofibroblasts are few in number, within the stroma of high-grade tumors, their number increases and are associated with a poor prognosis [7-9].

The most prominent stromal cells in tumors, termed "carcinoma-associated fibroblasts", are SMA-positive fibroblasts. For many years, research has focused on "carcinoma-associated fibroblasts" and their involvement in tumorigenesis, growth, and development of tumors. Although their role is still controversial, there is some evidence for their involvement in the initiation of tumor progression. For example, neoplastic transformation of breast epithelial cells has been observed with the overexpression of transforming growth factor β (TGFβ) and hepatocyte growth factor (HGF) in myofibroblasts [10].

Carcinoma-associated fibroblasts (CAFs) support tumor growth and the process of metastasis by providing growth factors and remodeling proteins of the extracellular matrix (ECM). They synthesize HGF and epithelial growth factor (EGF), which stimulate cell proliferation and block apoptosis [3]. CAFs-derived matrix metalloproteinases (MMPs), such as MMP-1, MMP-3, MMP-7, and MMP-11, disrupt the ECM and basement membranes, which is a crucial step in the transition from carcinoma in situ to invasive carcinoma [11,12].

CAFs also play an important role in blood vessel neogenesis in tumors. Pro-angiogenic factors, such as vascular endothelial growth factor (VEGF), fibroblast growth factor-2 (FGF2), and platelet-derived growth factors (PDGFs), are secreted by CAFs and mediate angiogenesis [13-16].

CAFs also express numerous pro-inflammatory cytokines and chemokines, as a result of which immune cells enter the tumor stroma and further interact with the tumor cells [17,18].

Fibroblast growth factor - 2 (FGF2, bFGF) belongs to the family of heparin-binding growth factors. It and its ligands have multiple effects on the body, both locally and systemically. It plays a central role in the body's development and homeostasis. FGF-signaling controls the growth, migration, and differentiation of numerous cell types, and aberrant FGF-signaling in many tumor types is an attractive therapeutic target as it affects multiple cells in the tumor microenvironment [19,20].

## Materials and methods

Study design

The present study was performed on formalin-fixed paraffin-embedded endoscopic biopsy specimens of early-stage (pTa and pT1) urothelial carcinomas of the urinary bladder, in a retrospective non-clinical manner, at a tertiary healthcare center - St. Marina University Hospital, Varna, Bulgaria.

Inclusion criteria were urothelial carcinoma of the urinary bladder diagnosed in our institution in the period 2007-11 in the aforementioned stages with a five-year follow-up period, while exclusion criteria were diagnosis at a separate healthcare institution for patients monitored at ours, presence of a higher stage malignancy, second histogenetically different malignancy, non-urothelial bladder carcinomas, or non-urinary bladder urothelial carcinoma. 

Ethical approval

All procedures carried out in the study fully adhered to the ethical standards of the Helsinki declaration of 1975 and its seventh revision in 2013, as well as the ethical standards of the Bulgarian Ministry of Healthcare and the Bulgarian Medical Association. The study received ethical approval, protocol number 54/19.05.2016, issued by the Medical University - Varna Committee on Scientific Ethics.

Preparation and interpretation of histological slides

An indirect immunoperoxidase method was used for immunohistochemical (IHC) analysis using a primary FGF2 antibody (catalog number 74412; Santa Cruz Biotechnology, Dallas, Texas, USA) and a DAKO mini KIT high-Ph visualization system (catalog number K8024; Aligent technology, Santa Clara, California, USA), an anti-polyvalent detection system for 3,3'-diaminobenzidine. All slides were compared with the original H&E stained slides used for the initial diagnosis, with IHC interpretation performed on an Olympus BX50 light microscope (Olympus Optical Co, Ltd., Shinjuku, Tokyo, Japan). H&E slides were reevaluated for tumor histologic type according to the World Health Organization and International Society of Urological Pathology 2022 criteria for urothelial malignancies and graded based on the 2018 American Joint Committee on Cancer guidelines.

The evaluation of the IHC reaction was performed by evaluating ten fields at 400x magnification for each case, with the reaction intensity and percentage of positive cells being assessed using a histo-score (H-score) using the following formula:

H-score = (0 x % of I0) + (1 x % of I1+) + (2 x % of I2+) + (3 x % of I3+)

The intensity (I) of the reaction was determined for each cell in the different fields ranging from negative (0) to weak (1), moderate (2), and intensive (3). Calculated in this way, the H-score varies from 0 to 300. The final score was dichotomously divided according to the median (determination of cut-off value); all cases above the median were reported as high expression levels and those below the median as low.

The resulting data from the IHC tests were compared with the tumor stage (invasion), degree of differentiation, progression-free survival, patient age, and gender.

Statistical analysis

Statistical analysis of the obtained results from IHC was carried out using the Microsoft Office Excel 2016 (Microsoft Corporation, One Microsoft Way, Redmond, Washington, US) software package and Statistical Package for the Social Sciences (SPSS) for Windows, Version 25.0 (IBM Corporation, Armonk, New York, US). Descriptive analysis was performed for determining statistical parameters including mean [μ(X)], standard deviation (SD), minimum (min), and maximum value (max).

Cross-tabulation and chi-square analysis were performed for significant differences in the frequency performance of category values. Statistical significance in the chi-square test was considered at p≤0.05. Correlation analysis was conducted to assess the relationship between the indicators examined and establish the interaction's strength. The degree of association between variables was defined as significant at r>0.5<r=0.7, large at 0.7<r=0.9, and extremely large at r>0.9 at p≤0.05. Kaplan-Meier test was conducted for progression-free survival, and the student's t-test was to compare quantitative and qualitative indicators and examine their differences. The resulting data was presented as an arithmetic mean, and SD for the groups studied. Receiver operating characteristic (ROC) curve analysis was done to determine the role of accuracy and specificity of the predictability of certain indicators. The value of the area under the curve was between 0.5 and 1.0. Complete separation of values by an indicator was done with a score above 0.75 or 75%.

In all analyses, a permissible level of significance of p<0.05 at a 95% confidence interval (CI) was assumed. The graphical and tabular presentation of statistics was performed in Microsoft Office 2016 (Microsoft Corporation).

## Results

A total of 163 patients fit the inclusion criteria without contradicting the excluded ones and were split into two groups based on their stage: pTa or pT1.

**Figure 1:**
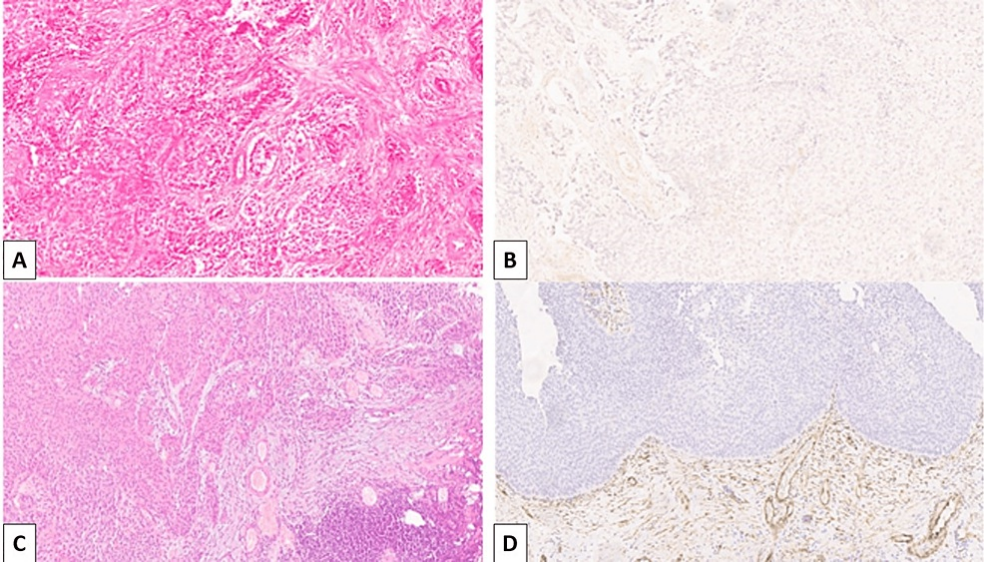
FGF2 expression in invasive and non-invasive urothelial carcinomas A: High-grade invasive urothelial carcinoma, H&E staining, original magnification x100; B: Lack of FGF2 expression in high-grade invasive urothelial carcinoma, original magnification x100; C: High-grade non-invasive urothelial carcinoma, H&E staining, original magnification x100; D: Moderately strong, diffuse cytoplasmic expression of FGF2 in high-grade non-invasive urothelial carcinoma, original magnification x100. H&E: hematoxylin and eosin; FGF2: fibroblast growth factor 2

The cytoplasmic expression of FGF2 was evaluated in the tumor stroma and gave a positive reaction in peri- and intratumoral fibroblasts of the stroma and blood vessel endothelial cells. The expression patterns (high and low) are shown in Figure 1.

**Figure 2:**
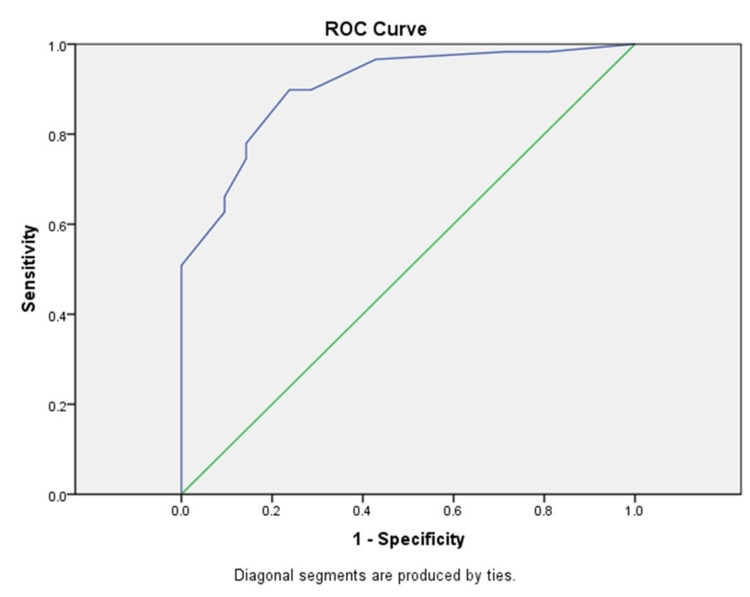
ROC curve analysis and determination of the cut-off value for FGF2 (AUC=0.904, 95% CI:0.834-0.975, p<0.001) ROC: receiver operating characteristic; FGF2: fibroblast growth factor 2; CI: confidence interval

Expression levels in stromal fibroblasts differed between the two groups of stage pTa and pT1 urothelial carcinomas. Using ROC curve analysis, we determined the cut-off value of 110 and divided FGF2 expression levels into low and high. The analysis showed that at optimal cut-off values of cytoplasmic expression in fibroblasts within the tumor stroma, the sensitivity is 75.4% and the specificity - 78.9% (area under the curve (AUC)=0.904, 95% CI: 0.834-0.975, p<0.001) (Figure 2).

ROC curve analysis showed that high levels of FGF2 expression in the stroma are associated with a low potential of urothelial carcinomas to infiltrate the submucosa (p<0.0001).

No further statistically significant results were derived from comparing the FGF2 stromal expression levels and the clinicopathological parameters of the patients, such as gender, age, differentiation, and occurrence of local recurrence.

## Discussion

The growth factor we studied, FGF2, shows significantly varying expression, based both on the percentage of expressing cells and cellular expression intensity when studied by means of IHC in early-stage urothelial bladder malignancies. This makes it a good candidate for further research, both based on its varying expression and biological role as a growth factor with variable cellular targets, along with the role of the stroma in tumor invasion and progression, as well as the results from the current study.

As seen in other conditions, FGF2 promotes a high mitogenic potential and is involved in many biological processes, including tumor growth and invasion [21-25]. It modulates the role of eosinophilic and mast cells and other immune cells, making it an extremely important factor in the progression of urothelial carcinomas, especially regarding the latter's role in them [23-25]. Its receptor (FGFR3) shows mutations and overexpression in a large proportion of urothelial carcinomas and even represents a target for therapy with tyrosine kinase inhibitors [26].

In our study, positive expression of FGF2 was detected by means of IHC in the cytoplasm of fibroblasts in the tumor stroma and around the tumor, as well as in the cytoplasm of endothelial cells from blood vessels. The present study found no relationship between FGF2 and differentiation/recurrence of urothelial carcinomas. However, there is a strong statistical correlation towards invasiveness in low expression levels.

Of similar importance for the invasiveness of urothelial carcinoma is the expression of cluster of differentiation (CD) 10 - a membrane metalloprotein with a very controversial role in the biological behavior of carcinomas of different localization [27,28]. Our previous research on another group of urothelial carcinomas of the bladder showed positive CD10 expression in tumors invading the underlying smooth muscle, while no expression was detected in non-invasive tumors [27]. As CD10 inactivates a number of ECM factors by cleaving amino-terminal peptide bonds and facilitating tumor progression, FGF in particular, it could be assumed that it is involved in blocking FGF-mediated angiogenesis and hence promoting the invasive potential of these neoplasms in a two-hit mechanism [28].

Study limitations and future direction

As our study involves a relatively small amount of urothelial neoplasms of the bladder for such a common clinical entity, future studies should be encouraged to evaluate its role in a larger cohort that also includes more invasive entities, pT2 and beyond. Furthermore, a more detailed study of the tumor-ECM interaction involving tumor-associated remodeling markers and ECM factors, such as CD10-FGF2 interaction, could show promise as an immunological profile of aggressive and invasive tumors and distinguish them from more indolent forms.

Furthermore, the tumor-EMC interactions are a factor for the development of metastasis, also unevaluated by our study, where it remains to be seen if FGF2 plays a crucial, if any, role.

## Conclusions

As seen by the result of our study, FGF2 expression in the ECM is directly associated with the invasive potential of early urothelial malignancies of the bladder. This indicates a broader tumor-EMC set of interactions, where the direct remodeling of the ECM by the tumor itself is not only predisposing but also indicative of invasion.

While it remains undetermined if such interactions also play a role in the metastatic potential of these neoplasias as a set of circumstances associated with the tumor-ECM interaction, future studies are encouraged to investigate the broader set of interactions and outcomes associated with these circumstances.
